# Supplementary material for: Molecular Characterization and Pathogenicity of an Infectious cDNA Clone of Youcai Mosaic Virus on Solanum nigrum
Source: Int J Mol Sci. 2024 Jan 28;25(3):1620. doi: 10.3390/ijms25031620 (PMC10855738; doi:10.3390/ijms25031620)
Supplement: Supplementary file 1 [file ijms-25-01620-s001.zip › Supplementary Figures.pptx]

## Slide 1
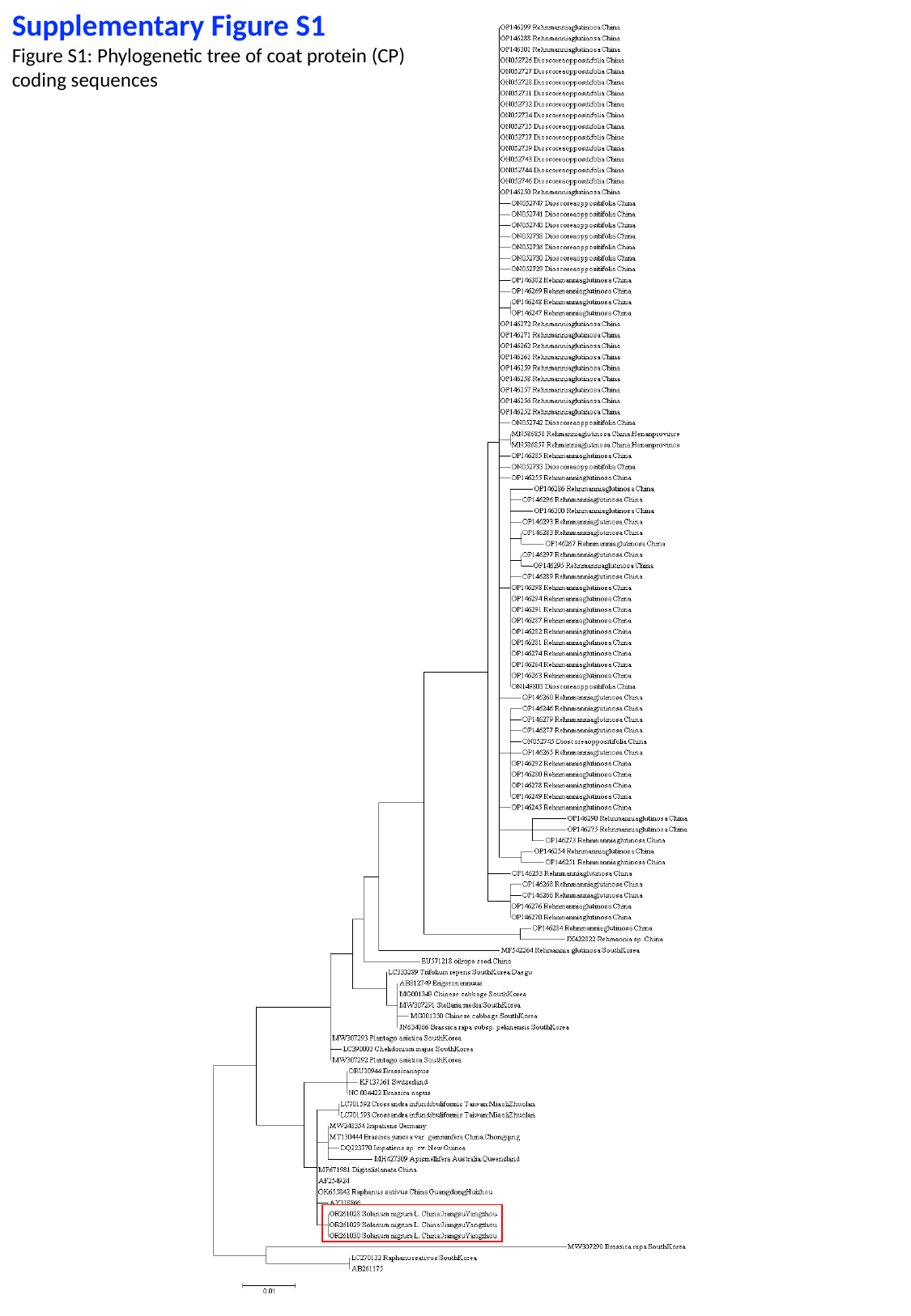

Supplementary Figure S1
Figure S1: Phylogenetic tree of coat protein (CP) coding sequences

## Slide 2
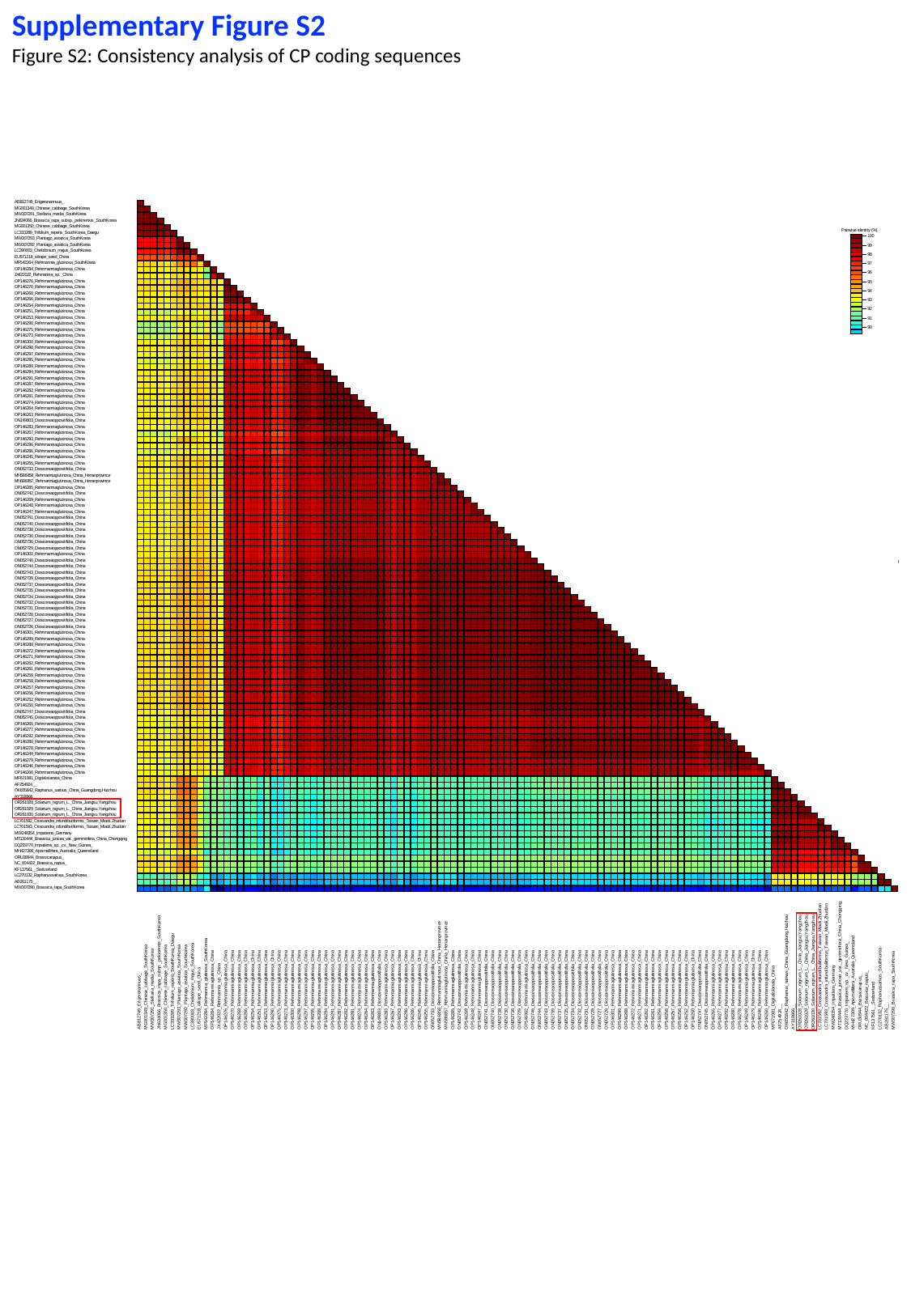

Supplementary Figure S2
Figure S2: Consistency analysis of CP coding sequences

## Slide 3
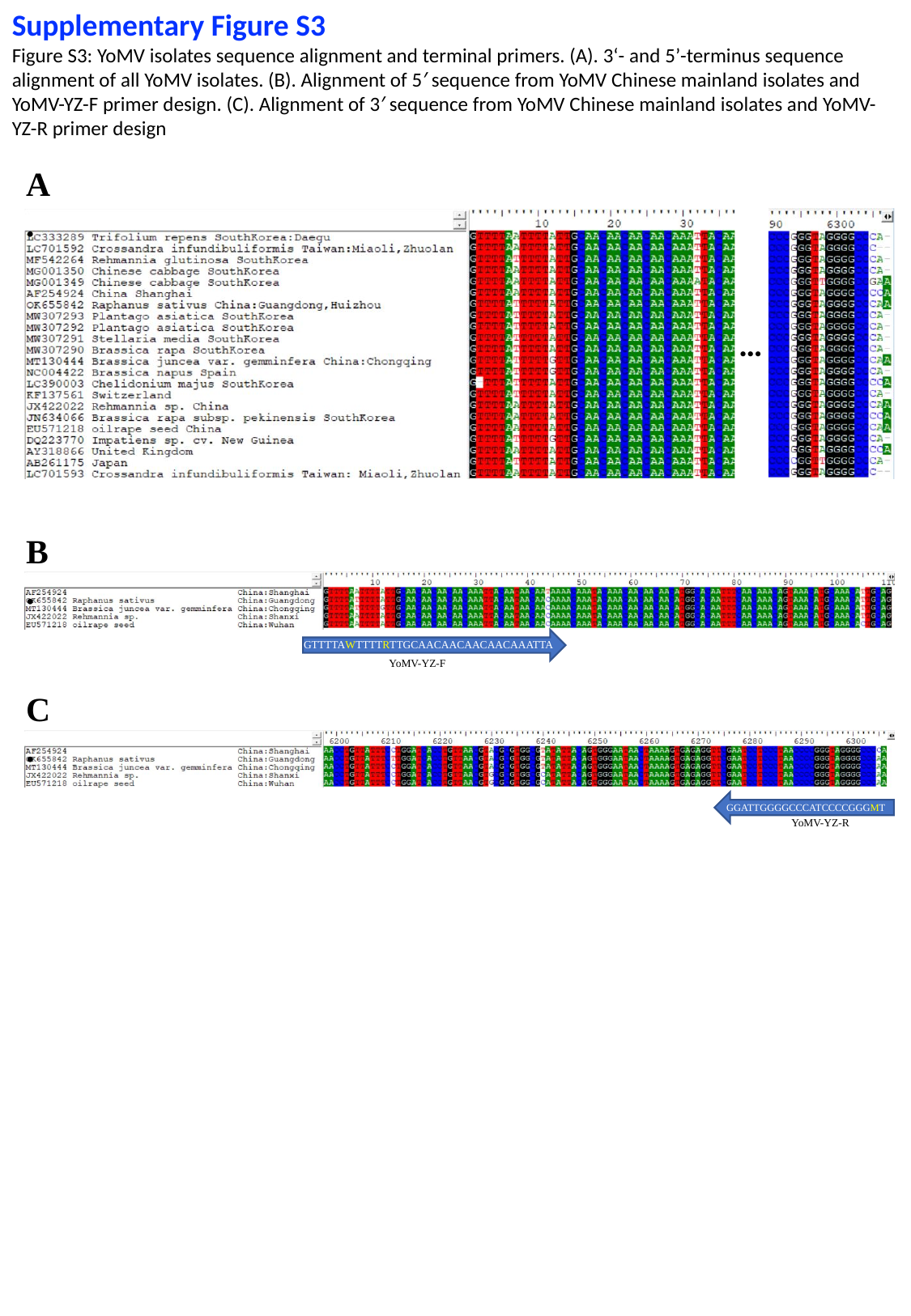

Supplementary Figure S3
Figure S3: YoMV isolates sequence alignment and terminal primers. (A). 3‘- and 5’-terminus sequence alignment of all YoMV isolates. (B). Alignment of 5′ sequence from YoMV Chinese mainland isolates and YoMV-YZ-F primer design. (C). Alignment of 3′ sequence from YoMV Chinese mainland isolates and YoMV-YZ-R primer design
A.
…
B.
GTTTTAWTTTTRTTGCAACAACAACAACAAATTA
YoMV-YZ-F
C.
GGATTGGGGCCCATCCCCGGGMT
YoMV-YZ-R
