# Supplementary material for: Molecular Characterization and Pathogenicity of an Infectious cDNA Clone of Youcai Mosaic Virus on Solanum nigrum
Source: Int J Mol Sci. 2024 Jan 28;25(3):1620. doi: 10.3390/ijms25031620 (PMC10855738; doi:10.3390/ijms25031620)
Supplement: Supplementary file 1 [file ijms-25-01620-s001.zip › Supplementary Table S1. Related viruses identified using the High-throughput siRNA deep sequencing.pdf]

Supplementary Table S1 Related viruses identified using the High-throughput siRNA deep sequencing

Virus name	Length of contigs/bp	Reference sequence	Percent Ident%
YoMV	2794	AF254924.1	99
YoMV	250	MG001350.1	99
YoMV	172	LC701593.1	98
YoWV	578	MF671981.1	99
TPCTV	137	NC_003825.1	90
