# Supplementary material for: Molecular Characterization and Pathogenicity of an Infectious cDNA Clone of Youcai Mosaic Virus on Solanum nigrum
Source: Int J Mol Sci. 2024 Jan 28;25(3):1620. doi: 10.3390/ijms25031620 (PMC10855738; doi:10.3390/ijms25031620)
Supplement: Supplementary file 1 [file ijms-25-01620-s001.zip › Supplementary Table S2. Pairs of primers used for amplification of different genes in this study.pdf]

Supplementary Table S2 Pairs of primers used for amplification of different genes in this study

Primer Name	Sequence (5'-3')	Length of the amplicon (bp)	Tm (℃)	Purpose
YoMV-YZ-F	GTTTTAWTTTTRTTGCAACAACAACAACAAATTA	3440	58	Amplification of the Frag.1 of YoMV
YoMV-YZ-R1	TGACTTAGGCGTCGGGACAAACAGATTC			
YoMV-YZ-F2	GAATCTGTTTGTCCCGACGCCTAAGTCA	2892	61	Amplification of the Frag.2 of YoMV
YoMV-YZ-R	TMGGGCCCCTACCCGGGGTTAGG			
pCA4Y-Frag1-F	CATTTCATTTGGAGAGGCCTGTTTTAATTTTATTG CAACAACAACAACAAATTAC	3460	64	Construction of pCA4Y-Frag.1 vector
pCA4Y-Frag1-R	GGATCCGATACCCTGTCACCTGACTTAGGCGTCG GGACAAACAGATTC			
pCA4Y-Frag2-F	CATTTCATTTGGAGAGGCCTGAATCTGTTTGTCCC GACGCCTAA	2912	68	Construction of pCA4Y-Frag.2 vector
pCA4Y-Frag2-R	GGATCCGATACCCTGTCACCTGGGGCCCCTACCC GGGGTTAGG			
YoMV-CP-F	ATGGTTTACAACATCACGAGCTCG	474	58	Amplification of the CP gene of YoMV
YoMV-CP-R	TGTAGCTGGCGCAGTAGCCCAAG			
TPCTV-CP-F	ATGCCTTACAAGCGGAAATTAACG	729	55	Amplification of the CP gene of TPCTV
TPCTV-CP-R	TTACGCATTTCCATTTCTATGATACATAG			
