# Supplementary material for: Molecular Characterization and Pathogenicity of an Infectious cDNA Clone of Youcai Mosaic Virus on Solanum nigrum
Source: Int J Mol Sci. 2024 Jan 28;25(3):1620. doi: 10.3390/ijms25031620 (PMC10855738; doi:10.3390/ijms25031620)
Supplement: Supplementary file 1 [file ijms-25-01620-s001.zip › Supplementary Table S3. Occurrence of YoMV on Solanum nigrum L. in different cities located in Jiangsu Province.pdf]

Supplementary Table S3 Occurrence of YoMV on Solanum nigrum L. in different cities located in Jiangsu Province

	Nanjing	Suzhou	Xuzhou	Yancheng	Nantong
Sample number	S23-S31	S32-S37	S38-S41	S42-S46	S47-S50
YoMV infected	S24, S25, S27, S29-S31	S33, S35, S36	S38, S39, S41	S43-S46	S47, S48, S49
YoMV infection rate	66.7%	50%	75%	80%	75%
